# Does Better Post-Thaw Motility of Dog Sperm Frozen with CLC Mean Better Zona Pellucida Binding Ability?

**DOI:** 10.3390/ani13101580

**Published:** 2023-05-09

**Authors:** Zuzanna Ligocka, Agnieszka Partyka, Sabine Schäfer-Somi, Anna Mucha, Wojciech Niżański

**Affiliations:** 1 Department of Reproduction and Clinic of Farm Animals, Faculty of Veterinary Medicine, Wroclaw University of Environmental and Life Sciences, Plac Grunwaldzki 49, 50-366 Wrocław, Poland; zuzanna.ligocka@upwr.edu.pl (Z.L.); agnieszka.partyka@upwr.edu.pl (A.P.); 2 Platform for Artificial Insemination and Embryo Transfer, University of Veterinary Medicine Vienna, Veterinärplatz 1, 1210 Vienna, Austria; sabine.schaefer@vetmeduni.ac.at; 3 Department of Genetics, Faculty of Biology and Animal Science, Wroclaw University of Environmental and Life Sciences, Kożuchowska 7, 51-631 Wrocław, Poland; anna.mucha@upwr.edu.pl

**Keywords:** canine, semen, membrane fluidity, cholesterol, capacitation, tyrosine phosphorylation, zona pellucida, oocyte

## Abstract

**Simple Summary:**

New cryopreservation methods for dog semen are constantly under investigation because the fertility results after insemination with frozen–thawed semen are still not satisfying. Most researchers do not investigate the fertilization ability of spermatozoa after the modification of cryopreservation protocols. In this study, we investigated whether spermatozoa membrane fluidity modification by use of cholesterol-loaded cyclodextrins (CLC) can improve kinematic parameters and also this method’s effects on capacitation status and fertilization ability. The lowest concentration of CLC increased motility parameters and percentage of live spermatozoa without cholesterol efflux compared to the control. There was no change in capacitation status but the binding ability of the sperm to the oocyte was decreased. Overall, these data suggest that improvement of kinematic parameters does not necessarily improve the zona pellucida binding ability results of canine spermatozoa.

**Abstract:**

Even though the search for methods improving cryopreservation of canine spermatozoa led to an improvement of post-thaw quality, fertilizing results after insemination with frozen–thawed semen are still not satisfying. In this study, we focused on modification of spermatozoa membrane fluidity and investigated whether kinematic parameters as assessed by computer-assisted semen analyzer (CASA) can be improved. The primary aim of our study was to investigate whether the use of cholesterol-loaded cyclodextrins (CLC; 0.5 mg, 1 mg, 2 mg) and 2-Hydroxypropyl-ß-cyclodextrin (HBCD; 1 mg) positively influence capacitation status as examined by tyrosinphosphorylation, cholesterol efflux and zona binding assay (ZBA) of spermatozoa. The use of 0.5 mg of CLC increased the percentage of motile, progressive and rapid spermatozoa compared to the control. Addition of HBCD decreased motility and progressive motility of spermatozoa and the population with rapid movement in comparison to the control. The percentage of live spermatozoa without efflux of cholesterol compared to the control was increased when extender with 0.5 mg of CLC was used. There was no change in capacitation status. The zona binding ability of spermatozoa was significantly lower in the group with 0.5 mg of CLC than in the control. In conclusion, these results suggest that improvement of kinematic parameters does not necessarily coincide with better zona pellucida binding ability of spermatozoa.

## 1. Introduction

In recent years, canine breeding has been constantly developing and dog breeders are learning more about reproduction techniques. This led to an increasing request for reproductive technologies in the small animal veterinary practices during recent years and motivates the search for new methods of canine semen cryopreservation. Frozen semen is of great interest in dog breeding for many reasons. It allows international shipment and exchange of the genetic pool in kennels and also storage of genetic material of the most valuable stud dogs. Constantly, new methods of freezing and storage of dog semen are used to obtain better results [1], but still the most frequently used method of cryopreservation of dog semen is freezing and maintenance in liquid nitrogen (LN2) at −196 °C. A lot of research has also been dedicated to various types of cryodiluents. The research included the use of 6% low-density lipoprotein (LDL) instead of egg yolk (EY), 1% soybean lecithin type IV-S instead of EY, dimethylformamide (DMF) instead of glycerol and some other additives to extenders such as metformin, ergothioneine and 3,4-dihydroxyphenyl glycol (DHPG) [2,3,4,5,6]. Most of these modifications improved or at least did not significantly change post-thaw motility in comparison to the control groups. Even though post-thaw semen quality is improving, fertilizing results are still not satisfying [7]. However, the success of artificial insemination is determined by many factors. Therefore, there is a problem in relating the composition of semen preservation extenders and freezing protocols with actual birthing results which is caused by, e.g., genetic factors, age and health and proficiency in determining the optimal day of, and technique for, the artificial insemination. It has been proven that the fertilization capability of semen should be assessed by using a combination of different tests to give reliable results [8,9]. Cryopreservation and thawing damage the acrosome and plasma membrane of spermatozoa [10,11,12]. Therefore, modification of plasma membrane fluidity is supposed to help sperm to survive low temperatures [13]. For the above-mentioned changes in the sperm plasma membrane, cholesterol-loaded cyclodextrin complexes (CLCs) positively affect their fluidity and, as a result, the quality of semen after thawing in other mammal species [14]. We expect similar effects with canine semen and negative results when only 2-Hydroxypropyl-ß-cyclodextrin (HBCD) is used as the acceptor for cholesterol. Cyclodextrins are cyclic oligosaccharide sugars that are capable of encapsulating hydrophobic compounds such as cholesterol and transporting them to the cell plasma membrane [15,16]. The complexes are assumed to prevent the rearrangement of phospholipids by increasing the cholesterol content of the membrane and thereby increasing membrane fluidity at low temperatures [17]. Because of these changes, it is important to examine the capacitation status of frozen–thawed spermatozoa. Physiologically, the regulation of sperm capacitation is regulated by an interaction between the sperm cell and the oviductal epithelium and fluids. Sperm capacitation is the sequence of membrane and intracellular events preceding the acrosome reaction in response to sperm interaction with the oocyte zona pellucida [18]. It has been proven in many animal species that addition of CLC can minimize cryocapacitation-like changes in sperm and improve the quality of frozen–thawed spermatozoa [14,15,19,20]. Another important test is the zona pellucida binding assay (ZBA) used to examine the fertilization ability of human [21] and animal [22,23] spermatozoa. Evaluation of the binding ability of spermatozoa to the zona pellucida is an important test to predict male fertility and supports routine sperm morphological evaluation to detect molecular damages on the membrane level [24]. Therefore, the aim of our study was to investigate the effect of CLC during semen freezing on the post-thaw kinematic changes, capacitation status and fertilizing ability of canine spermatozoa.

## 2. Materials and Methods

### 2.1. Reagents and Composition of Extenders

The Tris-citric acid–fructose–egg yolk extender (TFE) was composed of Tris (hydroxymethyl)-aminomethane (0.2 M), citric acid monohydrate (0.06 M), fructose (0.05 M), distilled water and 20% (*v*/*v*) of egg yolk [25]. This extender was used throughout the study and for cryopreservation was supplemented with different concentrations of cyclodextrins and cyclodextrin–cholesterol complexes. The extender was supplemented with 6% of glycerol for freezing [3]. 2-Hydroxypropyl-ß-cyclodextrin (HBCD) and cyclodextrin–cholesterol complex (CLC) were prepared according to the procedure adopted by Mocé et al. [14], and then the reagents were stored at 22 °C until use. The working solutions were prepared by adding 20 mg of CLC to 1 mL of TFE extender and stirring the solution using a vortex mixer. Canine capacitation medium (CCM) was prepared according to the procedure described by Mahi and Yanagimachi [26] and was used for the CASA and ZBA assessment after thawing. Propidium Iodide (1.0 mg/mL Solution in Water, P3566) was purchased from Thermo Fisher Scientific Inc. (Waltham, MA, USA) Other chemicals were obtained from Sigma-Aldrich (Merck KGaA, Darmstadt, Germany): Bodipy Cholesterol fluorophore (810255P), mouse anti-phosphotyrosine antibody (clone 4G10), Alexa Fluor 488-conjugated goat anti-mouse IgG (A32723), Dimethyl sulfoxide (DMSO) (D8418), Phosphate buffered saline (PBS) (P4417) and Bovine Serum Albumin (BSA) (A1470).

### 2.2. Animals and Semen Collection

Five Beagle stud dogs ranging in age from 3 to 6 years, owned by the Department of Reproduction and Clinic of Farm Animals in Wroclaw University (reg. 0057), were used in this study. The animals were in good health and reproductive condition. They were fed dry food once daily, with free access to water, and they had proper time of activity. The sperm-rich fraction of each dog was collected into a calibrated collection tube with water-coat pre-warmed to 37 °C. Then, ejaculates were preliminary checked under the microscope, qualified for the experiment and pooled into one semen sample. The semen was collected 9 times from each dog with 1 week apart and cryopreserved. Oocytes were recovered from ovaries collected from 18 bitches subjected to routine ovariohysterectomy. According to interview data with the owners, the females’ ages were 1.5–6 years and all of them were in anestrus at the day of ovariohysterectomy. All procedures were approved by the Local Ethical Committee in Wroclaw (statement n. 025/2020).

### 2.3. Cryopreservation and Thawing Method

Differences caused by individual semen quality and age of dogs [27] were limited by using pooled semen. The pool of semen was divided into five groups with different concentrations of CLC and one concentration of HBCD: (1) HBCD 1 mg/ 120 × 10^6^ cells, (2) CLC 0.5 mg/120 × 10^6^ cells, (3) CLC 1 mg/120 × 10^6^ cells and (4) CLC 2 mg/120 × 10^6^ cells. Each group was compared to the (5) control that stayed without any additions. All samples were centrifuged at 500× *g* for 5 min to remove seminal plasma and then extended with TFE with EY to a final concentration of 200 × 10^6^ spermatozoa/mL. In the next step, semen samples were cooled in the fridge to 5 °C within 1 h and then 6% of glycerol was added. Samples were equilibrated for 90 min at 5 °C. After equilibration, straws were filled and frozen at a temperature of −140 °C for 15 min in nitrogen vapor, 5 cm above the liquid nitrogen, and thereafter stored in liquid nitrogen [25]. After a few months of storage, the samples were thawed at 37 °C in a water bath for 60 s for detailed semen assessment.

### 2.4. Experiment 1

In experiment 1, the effect of different concentrations of CLC (0.5; 1; 2 mg) and HBCD (1 mg) on post-thaw sperm motility parameters, cholesterol efflux assay and tyrosine phosphorylation was studied.

#### 2.4.1. Computer-Assisted Semen Analysis

One straw from each freezing (100 × 10^6^ spz) was thawed and emptied in 2 mL of CCM. After thawing, the semen was centrifuged twice at 300× *g* for 5 min to remove the cryopreservation extender. The sperm pellet was rediluted in 2.5 mL of CCM (40 × 10^6^ spz/mL). Computer-assisted semen analyzer (CASA) Hamilton Thorne Sperm Analyser IVOS version 12.2l (Hamilton Thorne BioSciences, Beverly, MA, USA) was used to evaluate sperm suspension under 1.89 × 10 magnification. A three µL aliquot of semen was placed in a Leja analysis chamber (Leja, Nieuw-Vannep, The Netherlands) at 37 °C. Five fields were randomly selected. The following parameters were analyzed: the percentage of motile sperm (MOT), the percentage of progressively motile spermatozoa (PMOT), percentage of rapid spermatozoa (RAP), percentage of slow spermatozoa (SLOW), elongation (ELONG), straightness (STR), linearity (LIN), path velocity (VAP), progressive velocity (VSL), curvilinear line velocity (VCL), amplitude of lateral head displacement (ALH) and beat cross frequency (BCF). The analysis was performed with the following parameters: frame acquired 30, frame rate 60 Hz, minimum cell contrast 75, minimum cell size 4 pixels, straightness threshold 75%, path velocity threshold 100 µm/s^−1^ average path velocity (VAP) cut-off 9.0 µm/s^−1^, medium VAP cut-off 20 µm/s^−1^, head size non-motile 4 pixels, head intensity non-motile 80, static head size 0.44–4.98, static head intensity 0.49–1.68 and static elongation 17–96% [28].

#### 2.4.2. Cholesterol Efflux Assay

The procedure was performed as described by Bernecic et al. [29] with boar semen and by Schäfer-Somi et al. (2023) with dog semen. For this purpose, a stock solution of TopFluor^®^ Cholesterol was prepared (1 mM Bodipy in DMSO). Semen was thawed and washed 2 times by centrifugation using PBS (900× *g* for 5 min.). After washing, 50 µL of sample was taken for the control reaction and then 950 µL of PBS was added to the samples. A Bodipy Cholesterol fluorophore (810255P, Sigma-Aldrich, Vienna, Austria) was added at a final concentration of 0.4 mM to the samples and then incubated in a water bath at 37 °C for 10 min. After incubation, the semen was washed with 2 mL of PBS. For the analysis, 50 µL of the sample was diluted with 950 µL of PBS, and then 2.5 µL of PI (5 µg/mL) was added. The fluorescence was measured after 10 min of incubation. A BD FACSCANTO II flow cytometer (Becton Dickinson Biosciences) was used for analysis. Bodipy cholesterol and PI were excited at a wavelength of 488 nm to detect Bodipy cholesterol fluorescence at 530 nm and PI fluorescence at 585 nm. Four populations were obtained: Q1—non-viable cells (PI+) without cholesterol efflux, Q2—non-viable (PI+) cells with cholesterol efflux, Q—viable (PI−) alive cells with cholesterol efflux and Q4—viable cells (PI−) without cholesterol efflux. The four control samples (sperm without additives and sperm with DMSO, PI and Bodipy only) were evaluated first before each measurement. The FlowJo (Version 10, FlowJO, LLC) software was used for calculation of data and results were given as percentage live/dead cells with/without cholesterol efflux per group.

#### 2.4.3. Immunolocalization of Tyrosine-Phosphorylated (TP) Proteins in Spermatozoa

**Figure 1:**
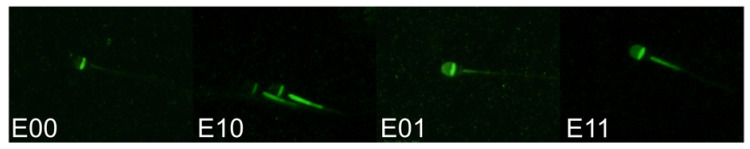
Class E00: non-phosphorylated sperm; class E10: spermatozoa showing tail protein tyrosine phosphorylation (midpiece or midpiece and additional fluorescence in endpiece); class E01: spermatozoa showing head protein tyrosine phosphorylation but without phosphorylation in the tail region; class E11: sperm with fluorescence in tail and head compartments [30].

Indirect immunofluorescence was used to visualize the localization of TP proteins in spermatozoa with the method described by Petrunkina et al. [30]. For tyrosine phosphorylation, the semen was taken from the same samples thawed for cholesterol efflux assay and washed two times using PBS (900× *g* for 5 min.). Air-dried smears were prepared using glass slides. Spermatozoa were fixed in 4% p-formaldehyde (PFA; *w*/*v*) for 30 min at room temperature (RT) and air dried. In the next step, spermatozoa were permeabilized using 0.5% Triton X-100 (*v*/*v*) for 15 min at RT. The slides were rinsed with PBS and incubated with 1% BSA in PBS (*w*/*v*; PBS-BSA) for 1 h at RT. They were incubated overnight at 4 °C with mouse anti-phosphotyrosine antibody (1:100 dilution; clone 4G10^®^; Merck Millipore, Darmstadt, Germany) in PBS-BSA. Prepared slides were washed three times with PBS-BSA. After washing, they were incubated for 1 h at RT with Alexa Fluor 488-conjugated goat anti-mouse IgG in the dark (1:100 dilution; Thermo Fisher Scientific, Breda, The Netherlands). After rinsing with PBS, spermatozoa were counterstained using DAPI [31] and extensive washed with PBS. Slides were mounted with Entellan (rapid mounting medium, 107960, Merck Millipore, Vienna, Austria) and a coverslip was put in place. A total of 200 cells was evaluated per sample under a fluorescence microscope at magnification ×1000 (oil immersion, filter cube U-MWB; Figure 1), and the percentages of each pattern were assessed.

### 2.5. Experiment 2

In experiment 2, the group with the best results in experiment 1 (CLC 0.5), regarding sperm motility parameters, was compared to the control group using a zona binding assay.

After the analyses performed in Experiment 1, where all 5 groups were tested, we decided to compare semen with 0.5 mg of CLC to the control group. One straw (100 × 10^6^ spz) from the selected groups was thawed at 37 °C for 60 s, and its content was emptied in 2 mL of CCM (post-thaw dilution rate 1:4). After dilution, the semen was washed by centrifugation twice at 300× *g* for 5 min to remove the cryopreservation extender. The sperm pellet was rediluted in 2.5 mL of CCM (40 × 10^6^ spz/mL). The sperm suspension was incubated at 38.5 °C and 5% CO_2_ in air for 24 h. After incubation, aliquots of spermatozoa were taken to analyse the zona binding capacity of the spermatozoa. In Petri dishes, 45 µL droplets of CCM were prepared and covered with 10 mL of mineral oil. The ovaries from females in the same age and stage of cycle, which were presented to routine ovariohysterectomy, were used. The ovaries were washed with PBS, frozen in 20 mL NaCl and then stored at –20 °C. In the day of experiment, ovaries were randomly chosen and thawed at room temperature. The cumulus−oocyte complexes were recovered by repeated slicing of the ovaries in a Petri dish with PBS and 0.5% (*w*/*v*) BSA under a stereomicroscope. Oocytes recovered and prepared using the procedure described by Ström Holst et al. [24] were stored overnight at 4 °C. The next day, 10 oocytes were pipetted into the droplets. The oocytes were incubated at 38.5 °C and 5% CO_2_ in air for 1 h [24]. A 25 µL aliquot of the incubated sperm suspension was diluted with 75 µL of CCM, and then a 5 µL aliquot of this dilution (containing around 25 × 10^3^ spz) was added to each droplet [24]. The droplets containing oocytes and spermatozoa were co-incubated at 38.5 °C and 5% CO_2_ in air for 6 h. After incubation, the oocytes were transferred to 100 µL droplets of glutaraldehyde (1.5%, *v*/*v*) in sodium cacodylate buffer (0.1 M) and fixed for 15 min. Thereafter, the sperm–oocyte complexes were washed by repeated pipetting in 100 µL droplets of PBS + BSA to remove loosely bound spermatozoa. After washing, oocytes were transferred to 100 µL droplets of a staining solution containing 10µL PI (Propidium Iodide—1.0 mg/mL Solution in Water, P3566, Thermo Fisher Scientific Inc.) in 1 mL of PBS + BSA for 10 min. Stained sperm–oocyte complexes were placed on a slide and overlaid with a coverslip supported by four droplets of a Vaseline–paraffin mixture. Pressure was applied to the four droplets to immobilise the oocytes in the CCM. The number of spermatozoa strongly bound to the zona pellucida was assessed using a fluorescent microscope equipped with laser of 488 nm excitation wavelength, operating at 100 mW. Observations were made at a magnification of 400×. Multiple optical two-dimensional sections at 10 m intervals were viewed and photos were performed for each oocyte. All photos from each evaluated oocyte layer were combined into one by using OLYMPUS cellSens Entry 1.18 software and then bounded spermatozoa were counted. The experiment was replicated 7 times for the control group and 6 times for the group with 0.5 CLC to assess at least 100 oocytes.

### 2.6. Statistical Analysis

Statistical analysis was carried out using the R 4.2.2 program [32].

Basic descriptive statistics of all the analysed variables was done using the pastecs library [33]. The Shapiro–Wilk test was used to verify a normal distribution and the F test to check the homogeneity of variances in the groups under consideration. In the case of a normal distribution and the assumed homogeneity of variance, the statistical significance of the examined parameter was verified by the *t*-test for dependent samples. Otherwise, the Wilcoxon test for related samples was used. This part of the analysis was carried out with the rstatix package [34]. The dependence analysis of the analysed variables was carried out using the Pearson correlation coefficient in the psych library [35]. The statistical significance of the correlation coefficients was verified at the level of 0.05. The statistical significance of the differences between the correlation coefficients in the considered groups was verified by analysing the confidence intervals. Corresponding correlation coefficients were considered statistically significantly different if their confidence intervals did not coincide.

## 3. Results

### 3.1. Experiment 1

All results obtained from analyses of cryopreserved canine semen with HBCD and different concentrations of CLC were compared to the control group. The sperm motility parameters assessed after thawing and washing with CCM are shown in Table 1. The percentage of motile, progressively motile and rapid spermatozoa were higher (*p* < 0.05) when addition of 0.5 mg CLC was used. Motility and progressive motility of spermatozoa and population with rapid movement were lower (*p* < 0.05) in the group with HBCD relative to the control. On the other hand, higher (*p* < 0.05) values for the linearity (LIN) and straightness (STR) of sperm movement were obtained in the group with 1 mg HBCD than in the control (Table 1). Sperm head elongation (ELONG) was lower (*p* < 0.05) in all experimental groups in comparison to the control. Addition of 2 mg of CLC and 1 mg of HBCD decreased (*p* < 0.05) beat cross frequency (Table 1). There were no significant differences in other parameters as assessed by CASA.

Cholesterol efflux from spermatozoa assessed by flow cytometry is shown in Table 2. The percentage of dead spermatozoa with cholesterol efflux decreased (*p* < 0.05) in the group with 2 mg of CLC. At the same time, the percentage of dead spermatozoa without efflux of cholesterol increased (*p* < 0.05) in the same group (2 mg of CLC) in comparison to the control. Addition of 0.5 mg of CLC increased (*p* < 0.05) the percentage of live spermatozoa without efflux of cholesterol compared to the control.

Sperm capacitation levels described by immunolocalization of TP protein in spermatozoa are shown in Table 3. The following types of phosphorylation were differentiated: non-phosphorylated sperm; tail-phosphorylated cells (midpiece phosphorylation or entire-tail phosphorylation) without head fluorescence; head fluorescence without tail fluorescence and head fluorescent with different degrees of tail fluorescence (Figure 1). Spermatozoa showing head protein tyrosine phosphorylation but without phosphorylation in the tail region (class E01) was the most-often (except class E00) noticed for all tested groups. Results from all experimental groups in comparison to the control did not change significantly (Table 3).

### 3.2. Experiment 2

**Figure 2:**
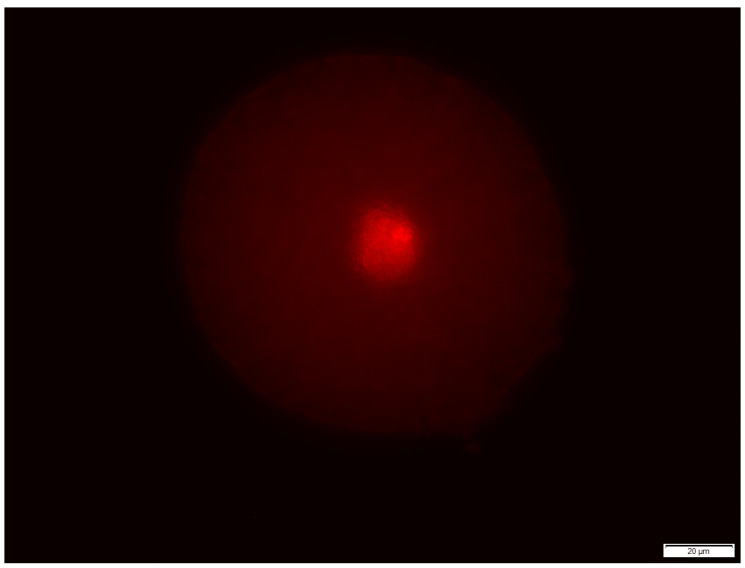
Zona binding assay–oocyte without bound spermatozoa pellucida (PI staining).

**Figure 3:**
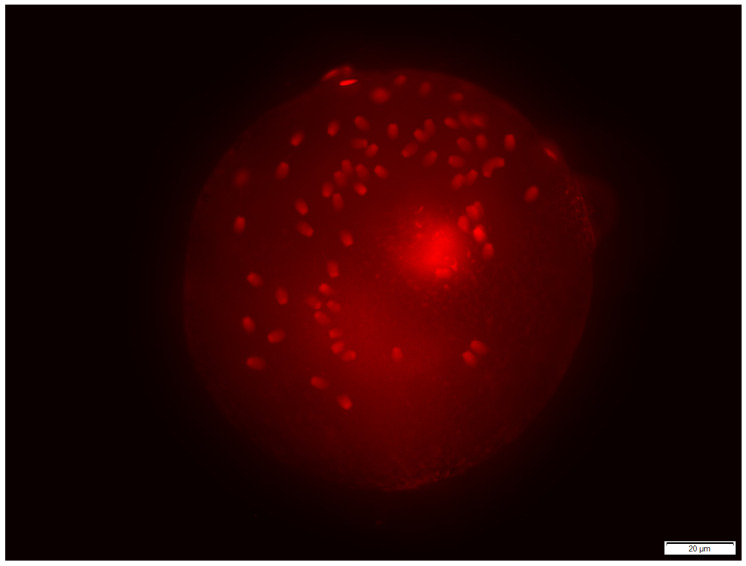
Zona binding assay–oocyte with many spermatozoa bound to the zona pellucida (PI staining).

Zona binding assay results are shown in Table 4. The sperm-binding capacity of the oocytes in both groups (control and 0.5 mg of CLC addition) differed between the replicates with a *p*-value in the control group of *p* < 0.001 and with a *p*-value in the group with CLC of *p* < 0.001. Significantly more spermatozoa bound to the zona pellucida when using extender without addition (control) than when 0.5 mg of CLC was used, at *p* < 0.001 (Table 4). In both groups, there were oocytes without any bound spermatozoa (Figure 2) and some with many spermatozoa attached (Figure 3).

## 4. Discussion

Canine semen freezability and fertilizing results after insemination with frozen–thawed semen are still not satisfying [7]. It has been proven that the fertilization capability of semen should be assessed by combination of different tests to increase the reliability of results [8,9]. This is the reason why we decided to extend basic semen analysis with the assessment of capacitation status and the ability of sperm to bind to the zona pellucida of oocytes [24]. In this study, we compared the impact of low concentrations of CLC (0.5; 1.0; 2.0 mg per 120 × 10^6^ sperm) and 1.0 mg of 2-hydroxypropyl-β-cyclodextrin (HBCD) on frozen–thawed canine spermatozoa. Kinematic data were assessed by the CASA system after washing the semen twice in CCM. Motility parameters obtained in our experiment were lower than described by other authors [2,4,36,37]. It is well known that centrifugation decreases motility parameters of spermatozoa, especially after cryopreservation [38], and centrifugation twice before the measurements could be the reason of the low results of MOT, PMOT and RAP. However, with this manipulation, the percentage of spermatozoa with progressive movement in groups with CLC was higher than it was shown by Inanc et al. [39], who analysed the semen directly after thawing. Our study confirms the more effective preservation of sperm cell function after cholesterol insertion into the cell membrane on the basis of the significantly higher number of motile cells, cells with progressive movement and rapid cells obtained with spermatozoa cryopreserved in the extender with 0.5 mg of CLC. Extender with addition of 1 mg of CLC had no impact on the sperm features evaluated by CASA. The best concentration of cholesterol-loaded cyclodextrins was 0.5 mg, and this result confirms similar findings in stallions [19], gazelles [40] and bulls [41]. It has been reported that other concentrations such as 0.75 mg, 1 mg and 1.5 mg of CLC also had a positive effect on motility parameters in rams [14], stallions [42] and bulls [43]. Another important finding was that even small concentrations of HBCD significantly decreased MOT, PROG and rapid cells, which corresponds to the results of other authors [20]. Addition of 2 mg of CLC to the diluent decreased canine sperm beat cross frequency (BCF) in a similar way as HBCD, but the other kinematic parameters did not change significantly. Interestingly, in rams and bulls, addition of 2 mg of CLC to the diluent had a positive impact on sperm motility [14,44], showing that the effect of CLC is not only dose-dependent but also species-specific.

The ATP-binding cassette (ABC) transporters are membrane molecules regulating the cholesterol and phospholipid transport within and through cell membranes of many different cells, including spermatozoa [45,46]. Expression of the ABC-transporter A1 molecule (ABCA1) was mainly detected in the acrosome and midpiece in the membranes of canine epididymal and ejaculated spermatozoa [47]. The TOPFLUOR^®^ Cholesterol kit (Avanti Polar Lipids, Alabaster, AL, USA) is a quite new tool and can be used to measure cholesterol efflux in variable cells [48,49,50]. Recently, it was proven that the cholesterol balance can also be assessed in mammalian spermatozoa [29,51]. In studies conducted on dog spermatozoa, different concentrations of probucol were assessed for inhibition of ABCA1. Probucol is a lipid-lowering drug which was found to significantly inhibit cholesterol efflux in human skin fibroblasts and J774 mouse macrophages. The study clearly showed that, in dogs, the impact of ABCA1 on cholesterol efflux is high and can be inhibited to a certain extent in a dose-dependent manner by probucol [52]. Our study showed that 0.5 mg of CLC increased the percentage of live spermatozoa without cholesterol efflux. Our results proved that cholesterol within cholesterol-loaded cyclodextrin complex (CLC) can successfully be transported into the cell plasma membrane without stimulation of further cholesterol efflux.

The mechanism of tyrosine phosphorylation of dog sperm membrane proteins is not yet fully understood, but it is known that it occurs during the first stages of capacitation [53]. One study showed the dependence of tyrosine phosphorylation on glucose and fructose [54], but this result was not fully confirmed. The tyrosine phosphorylation of canine sperm after induction of capacitation was investigated by Petrunkina et al. [30,53] The authors found that, as capacitation progressed, sperm proteins of different compartments became gradually phosphorylated over time [53]. Despite the fact that CLC and HBCD had an influence on cholesterol content in plasma membranes, our study confirmed that addition of these substances in proposed concentrations did not affect the capacitation status of sperm.

Sperm binding to the zona pellucida of oocytes is a very complex process [55] and the plasma membrane of the spermatozoa is a crucial element of this mechanism [56]. On the plasma membrane of the anterior head, receptors for the glycoproteins that compose the zona pellucida are localized [57]. One of the glycoproteins, ZP3, stimulates a signal transduction pathway in sperm that leads to the exocytosis of the acrosome (acrosome reaction), helping sperm to penetrate the zona pellucida [57]. Zona pellucida binding assay (ZBA) was used to evaluate the ability of spermatozoa to bind to oocytes in several species [58,59]. The ZBA can reveal functional impairment which may be missed when a routine sperm morphological evaluation is used [24]. It was proven that the binding of dog spermatozoa to the zona pellucida is a specific feature of living cells and is not influenced to any great extent by, for example, the stickiness of the zona pellucida [24]. In cattle, a correlation between zona pellucida binding ability and fertility was found [22]. On the other hand, in boars, the in vitro interaction was not found to be related to fertility [60], nor was it found to be a useful indicator of the fertilization capacity of frozen–thawed ram spermatozoa [61]. The low correlation between ZBA outcomes and field fertility observed in some species may be partially explained by existence in the sample of different subpopulations of motile spermatozoa. The mentioned correlation was considered by Peña et al. [62]. The outcome of a ZBA depends on many factors: the species, the sperm population and the quality of oocytes. Therefore, although interaction between the spermatozoon and the zona pellucida is essential, the ZBA is not useful for prediction of male fertility in all cases and all species. The importance of the ZBA increases considering the use of sperm for in vitro fertilization, a method with very low success rates in dogs [63,64]. In dogs, differences in the sperm-zona pellucida binding ability reveal malfunctions in spermatozoa and also damages caused by freezing-thawing of spermatozoa, causing a decrease in fertility [65]. In our study, even though, after thawing, there were no differences in the capacitation levels between groups, there were significantly less spermatozoa bound to the ZP in the group with CLC than in the control group. Even though Peña et al. [66] found that motile and viable frozen–thawed canine spermatozoa lose their ability to bind to the ZP after capacitation, this cannot be the reason for the observed differences in this study. In dogs, the ZBA with frozen–thawed sperm may reveal variable numbers of bound spermatozoa per oocyte between replicates [66,67], which is in agreement with our results. Furthermore, it is known that, in humans and dogs, the quality of oocytes has a large impact on the number of bound spermatozoa [68,69]. In our studies, we used oocytes from bitches ranging between 1 and 6 years of age and collected from ovaries from bitches ovariohysterectomized in anestrus; nevertheless, the observed differences between groups might be the effect of variable oocyte quality. The stage of oocyte nuclear maturation has no effect on spermatozoa penetration and immature oocytes can be penetrated by spermatozoa [70]. Another point that is worth consideration, which may affect the differences in the number of attached spermatozoa between groups, is changes in the cholesterol content of the cell membrane of spermatozoa frozen with CLC. As was discussed above, the mechanism of sperm fusion with the zona pellucida of the oocyte is closely related to the composition of the sperm cell membrane [56,57]. The different results of the ZBA may be affected by changes in the acrosome reaction (AR) between the groups. The acrosome reaction has been associated with binding and penetration through the ZP [71]. Noteworthy is the fact that, in our study, in both groups, the mean number of bound spermatozoa was higher than described by other authors [24,67,72], and in all replications there were oocytes with bound spermatozoa. The answer to the question posed in the title does not seem to be obvious, due to the fact that it is not clear which is more important, the number of motile sperm being able to migrate through the utero-tubal junction or their ability to bind to the ZP [62].

## 5. Conclusions

In conclusion, we could show that defined concentrations of CLC improved post-thaw kinematics of canine spermatozoa. However, a concentration of 0.5 mg in the diluent decreased the in vitro zona binding ability of spermatozoa as proven by ZBA. This in vitro effect might not reveal the true fertilization capability of the treated semen samples, which still has to be proven in vivo.

## Figures and Tables

**Table 1:** The effect of HBCD and CLC on the sperm motility parameters of frozen–thawed canine semen after two washings with CCM (*n* = 9, mean ± SD).

	MOT [%]	PMOT [%]	RAP [%]	SLOW [%]	ELONG [%]	STR [%]	LIN [%]	VAP [µm/s]	VSL [µm/s]	VCL [µm/s]	ALH [µm/s]	BCF [Hz]
control	26.2 ± 10.2 ^a^	16.0 ± 7.9 ^a^	16.8 ± 8.6 ^a^	24.7 ± 9.9 ^a^	62.7 ± 3.2 ^a^	91.1 ± 2.4 ^a^	65.8 ± 5.5 ^a^	115.7 ± 11.3 ^a^	107.1 ± 10.2 ^a^	165.6 ± 23.92 ^a^	6.9 ± 1.1 ^a^	31.4 ± 2.5 ^a^
0.5 mg CLC	36.6 ± 12.8 ^b,^*	24.3 ± 10.1 ^b,^*	25.9 ± 10.8 ^b,^*	21.9 ± 12.3 ^a^	59.6 ± 2.2 ^b,^*	90.1 ± 2.2 ^a^	64.3 ± 5.9 ^a^	126.9 ± 17.0 ^a^	115.8 ± 16.1 ^a^	185.5 ± 25.7 ^a^	7.6 ± 1.3 ^a^	29.2 ± 3.0 ^a^
1 mg CLC	18.7 ± 13.0 ^a^	12.3 ± 9.4 ^a^	12.8 ± 9.8 ^a^	22.4 ± 15.6 ^a^	60.4 ± 2.2 ^b,^*	90.8 ± 1.9 ^a^	62.9 ± 4.2 ^a^	123.6 ± 21.5 ^a^	114.0 ± 19.5 ^a^	184.6 ± 41.0 ^a^	7.5 ± 1.4 ^a^	29.3 ± 2.0 ^a^
2 mg CLC	21.6 ± 12.0 ^a^	13.4 ± 10.3 ^a^	13.8 ± 10.7 ^a^	22.2 ± 15.2 ^a^	60.0 ± 1.6 ^b^	91.2 ± 2.1 ^a^	67.2 ± 4.5 ^a^	108.6 ± 16.3 ^a^	100.7 ± 14.0 ^a^	152.5 ± 27.8 ^a^	6.9 ± 0.8 ^a^	28.4 ± 1.8 ^b^
1 mg HBCD	13.4 ± 6.8 ^b^	6.6 ± 4.6 ^b^	6.6 ± 4.6 ^b^	21.0 ± 11.7 ^a^	59.1 ± 2.2 ^b^	90.6 ± 3.3 ^a^	65.0 ± 7.5 ^a^	109.7 ± 22.3 ^a^	100.8 ± 20.4 ^a^	159.1 ± 36.2 ^a^	7.2 ± 1.5 ^a^	28.7 ± 2.9 ^b^

(MOT) percentage of motile spermatozoa, (PMOT) percentage of progressively motile spermatozoa, (RAP) percentage of rapid spermatozoa, (SLOW) percentage of slow spermatozoa, (ELONG) elongation, (STR) straightness, (LIN) linearity, (VAP) path velocity, (VSL) progressive velocity, (VCL) curvilinear line velocity, (ALH) amplitude of lateral head displacement, (BCF) beat cross frequency. Given *p*-value is for a paired *t*-test (T) or for a paired Wilcoxon test (W) in comparison to the control group. Different superscripts in columns indicate significant differences: ^a,b^ *p* < 0.05, * one-sided test.

**Table 2:** The effect of HBCD and CLC on cholesterol efflux (Bodipy cholesterol) of frozen–thawed canine semen (*n* = 9, mean ± SD).

	Dead Sperm Effluxed [%]	Dead Sperm Non-Effluxed [%]	Live Sperm Effluxed [%]	Live Sperm Non-Effluxed [%]
control	42.1 ± 10.4 ^a^	41.1 ± 14.7 ^a^	8.9 ± 7.2 ^a^	8.0 ± 5.2 ^a^
0.5 mg CLC	46.5 ± 8.3 ^a^	36.0 ± 7.9 ^a^	7.5 ± 4.3 ^a^	10.0 ± 6.0 ^b,^*
1 mg CLC	43.4 ± 10.2 ^a^	40.6 ± 11.9 ^a^	7.9 ± 5.8 ^a^	8.1 ± 3.2 ^a^
2 mg CLC	37.2 ± 8.4 ^b^	45.9 ± 10.7 ^b,^*	8.3 ± 4.8 ^a^	8.7 ± 4.4 ^a^
1 mg HBCD	43.5 ± 8.3 ^a^	40.2 ± 11.4 ^a^	9.1 ± 5.8 ^a^	7.2 ± 5.3 ^a^

Given *p*-value is for a paired *t*-test (T) or for a paired Wilcoxon test (W) in comparison to the control group. Different superscripts in columns indicate significant differences: ^a,b^ *p* < 0.05, * one-sided test.

**Table 3:** The effect of HBCD and CLC on the sperm capacitation level described by immunolocalization of TP protein (Indirect immunofluorescence), (*n* = 9, mean ± SD).

	E00 [%]	E10 [%]	E01 [%]	E11 [%]
control	64.2 ± 15.3 ^a^	5.6 ± 4.6 ^a^	28.8 ± 14.2 ^a^	1.4 ± 2.5 ^a^
0.5 mg CLC	63.9 ± 13.4 ^a^	7.2 ± 3.8 ^a^	27.5 ± 11.3 ^a^	1.3 ± 1.4 ^a^
1 mg CLC	62.4 ± 10.5 ^a^	4.6 ± 1.9 ^a^	31.8 ± 9.0 ^a^	1.2 ± 1.2 ^a^
2 mg CLC	69.4 ± 9.3 ^a^	5.4 ± 3.0 ^a^	23.6 ± 8.4 ^a^	1.6 ± 1.5 ^a^
1 mg HBCD	66.8 ± 13.4 ^a^	4.9 ± 2.9 ^a^	27.5 ± 14.2 ^a^	0.7 ± 1.1 ^a^

Class E00: non-phosphorylated sperm; class E10: spermatozoa showing tail protein tyrosine phosphorylation (midpiece or midpiece and additional fluorescence in endpiece); class E01: spermatozoa showing head protein tyrosine phosphorylation but without phosphorylation in the tail region; class E11: sperm with fluorescence in tail and head compartments [33]. Given *p*-value is for a paired *t*-test (T) or for a paired Wilcoxon test (W) in comparison to the control group. Different superscripts in columns indicate significant differences: ^a^ *p* < 0.05.

**Table 4:** Number of canine spermatozoa bound to the zona pellucida of oocyte; comparison between the control and the group with 0.5 mg of CLC.

Group	Replicate	Number of Oocytes	Median (Range)	Mean ± SD	*p*-Value of the Replicate Effect Test (Effect Size)	*p*-Value of the Group Effect Significance Test (Effect Size)
control	1	11	27 ^a^ (1–89)	37.45 ± 26.53	4.43 × 10^−8^ (0.359 large)	7.81 × 10^−14^ (0.499 moderate)
	2	10	15 ^a^ (4–73)	28.10 ± 26.21		
	3	23	3 ^b^ (0–12)	3.83 ± 4.28		
	4	15	18 ^a^ (1–65)	19.73 ± 18.46		
	5	21	17 ^a^ (2–66)	18.76 ± 17.43		
	6	19	29 ^a^ (1–122)	36.63 ± 30.51		
	7	17	37 ^a^ (4–91)	36.88 ± 23.26		
	total	116	17.5 (0–122)	24.09 ± 24.33		
0.5 mg CLC	1	20	5 ^a^ (0–26)	8.55 ± 8.44	7.72 × 10^−5^ (0.209 large)	
	2	24	0.5 ^b^ (0–3)	0.92 ± 1.06		
	3	20	3.5 ^a^ (0–26)	7.20 ± 7.70		
	4	15	2 ^a^ (0–17)	5.67 ± 5.91		
	5	20	5 ^a^ (1–31)	6.65 ± 7.03		
	6	9	2 ^ab^ (0–9)	2.89 ± 3.33		
	total	108	2 (0–31)	5.38 ± 6.74		
total		224	7 (0–122)	15.07 ± 20.37		

Replicates differing statistically significantly are marked with different letters: ^a,b^ *p* < 0.01 in a Kruskal—Wallis test. *p*-value of the group effect significance test is for a Wilcoxon test for independent samples.

## Data Availability

The data presented in this study are available on request from the corresponding author.
